# The association between family adaptability and adolescent depression: the chain mediating role of social support and self-efficacy

**DOI:** 10.3389/fpsyg.2024.1308804

**Published:** 2024-03-26

**Authors:** Yanyan Lin, Guangyunxian Jia, Zirong Zhao, Meng Li, Guanghai Cao

**Affiliations:** ^1^College of Teacher Education, Jining University, Qufu, China; ^2^College of Education, Qufu Normal University, Qufu, China

**Keywords:** family adaptability, social support, self-efficacy, depression, adolescents

## Abstract

**Objective:**

Previous research has shown a correlation between family adaptability and adolescent depression. However, there is a lack of studies that have investigated the underlying mechanism between family adaptability and adolescent depression. Based on the Ecological Systems Theory, this study aims to investigate the link between family adaptability and depression in adolescents, mediated by the sequential roles of social support and self-efficacy.

**Methods:**

The sample consisted of 1086 students randomly selected from seven public middle schools in Shandong Province, Eastern China. All the participants filled in the structured self-report questionnaires on family adaptability, social support, self-efficacy, and depression. The data were analyzed using SPSS 25.0 and Structural Equation Modeling (SEM) in AMOS 24.0.

**Results:**

The findings of this study are as follows: (1) Family adaptability is negatively associated with adolescent depression; (2) Social support plays a mediating role between family adaptability and adolescent depression; (3) Self-efficacy plays a mediating role between family adaptability and adolescent depression; (4) Social support and self-efficacy play a chain mediation role between family adaptability and adolescent depression.

**Conclusion:**

It is suggested that early interventions and support should be provided to facilitate adolescents’ family adaptability, social support, and self-efficacy, thus reducing their depression and improving mental health of adolescents.

## Introduction

Recent data from the China Mental Health Survey indicates that the group of depressive disorder incidence in China gradually tends to be younger, with 30.28% of the incidence under the age of 18 (National Depression Blue Book, 2022). Depression is an important emotional manifestation of depressive disorders. If this emotion is intervened in a timely manner early in its emergence, it can significantly reduce an individual’s risk of developing depressive disorders. The word “depression” used in this study is a depressive mood. Depression is a common negative emotion among adolescents (Miller et al., 2023; Schwartzman et al., 2023; Wang et al., 2023). It is considered a common intrinsic maladaptive response (Chi et al., 2020), characterized by a non-specific period of sadness, unhappiness, or emotional distress in response to adverse situations or events in daily life and learning (Yang et al., 2000). Adolescent depression is associated with many factors. Evidence suggests that the lifestyle, stress, and trauma that adolescents encounter during their developmental careers can increase their risk of developing depressive disorders (Fox et al., 2010; Yin et al., 2022; Zhang et al., 2024). Studies have shown that parent–child, teacher-student, and peer relationships are negatively correlated with depression in adolescents (Bradford et al., 2017; Cao et al., 2023; Hasty et al., 2023). The better the relationship, the lower the risk of depression in adolescents. It is easy to see how depression affects adolescents not only in terms of academics, relationships, and social adjustment, but also as a predictor of their mental health (McLeod et al., 2016). In the short term, depression can negatively impact adolescents’ academic performance, social functioning, and interpersonal relationships (Chavez et al., 2023). In the long term, adolescents are prone to self-injury, suicide, and other behavioral problems once they are depressed (Ding et al., 2022; Neslusan et al., 2023), and at the risk of developing related psychiatric disorders in adulthood (Robin et al., 2017). Therefore, it is particularly important to improve the mental health level of adolescents, to prevent and reduce adolescent depression.

### Family adaptability and adolescent depression

Family adaptability refers to the capacity of a family system to adjust and respond to the changing circumstances and challenges that arise throughout different stages of family development (Olson, 1989). It is considered a crucial component in assessing the overall functioning of the family unit. Ecological Systems Theory suggests that an individual psychological development is the result of the interaction of environmental and individual factors (Bronfenbrenner and Ceci, 1994). In terms of environmental factors, the family is the most direct environmental system for the individual, and the development of individual behavior is closely related to factors such as emotional connection and communication patterns among family members (Bronfenbrenner, 1986). Relevant empirical studies have provided evidence supporting the significant predictive role of family adaptability in relation to adolescent depression (Ruiz-Robledillo et al., 2019; Liu X. et al., 2020). These studies have consistently shown that family adaptability negatively predicts levels of depression among adolescents. The findings from Nam et al. (2016), Xu and Zhang (2018), and Huang et al. (2022) further support this assertion, indicating that higher levels of family adaptability are associated with lower levels of depression in the adolescent population.

The study by Freed et al. (2016) yielded similar findings, indicating that higher levels of family adaptability in adolescents were associated with a lower likelihood of experiencing depression (Freed et al., 2016). Individuals who come from families with high levels of adaptability tend to receive various positive behavioral influences and emotional support, which serve as protective factors against adverse behaviors and promote positive behaviors. Consequently, the risk of developing depression is reduced (Jia and Yuan, 2018). Wang Enna et al. conducted a 3-year longitudinal follow-up of 1,301 Chinese students in grades 7–9 year, and the results showed that the association between family adaptability and adolescent depression was dynamic and time-dependent (Wang et al., 2021). The correlations and regression analyses of family adaptability and adolescent depression have only been discussed in existing studies, and the mechanisms of the two have not been investigated, such as the study by Li et al. (2021) There is a gap in the literature describing the mechanisms of family adaptability and depression, so the present study wanted to revalidate the relationship between the two and address the previous limitations. Drawing upon the aforementioned studies, it is evident that family adaptability exerts a substantial influence on adolescent depression. Adolescents with higher levels of family adaptability are more likely to be positive and confident in life (Daniel et al., 2020). Conversely, adolescents with lower levels of family adaptability are more likely to be pessimistic and backward in life, lose confidence, and increase their risk of depression (Lee et al., 2017). Based on this view, the following hypothesis is proposed:

*H1*: Family adaptability is negatively associated with adolescent depression.

### Social support as a mediator

Social support is the emotional, psychological, physical, informational, instrumental, and material assistance provided by others to maintain one’s health or to facilitate one’s adaptation to difficult life events (Dunst et al., 1986). Ecosystem theory suggests that the environment to which adolescents are exposed is not limited to the family, but peers and teachers in school also play important roles in their psychological development (Teresa et al., 2021; Adekunle et al., 2022), reflected primarily in social support. There is a discrepancy between the social support objectively provided by the outside world and the social support subjectively perceived by adolescents. Therefore, Zimet proposes the concept of perceived social support, and all references to “social support” in this study refer to perceived social support. Adolescents perceive social support as a subjective experience of support, which is the emotional experience and satisfaction of the individual himself/herself feeling respected, supported, and understood (Wataru et al., 2020; Raymond and Gou, 2022). Zimet classified perceived social support into three dimensions, namely family support, friend support, and other support (leaders, relatives, co-workers) (Zimet et al., 1988). According to the main effect model of social support, social support has a general gaining effect (Cohen and Wills, 1985). Studies have shown that social support can reduce individuals’ dysphoria (e.g., feelings of loneliness, anxiety, depression, etc.) (Eva et al., 2014; Yu et al., 2022), and individuals who receive more social support have lower levels of depression (Banks and Weems, 2014; McKillop et al., 2017). In other words, increasing social support can effectively reduce the level of depression (Yu et al., 2016). Social support is beneficial in relieving the individual’s stress and reducing their tendency for depression to occur (Kong et al., 2013; Chris et al., 2022). Social support was found to increase individuals’ positive experiences, enhance their self-evaluation, improve their positive self-concept, reduce loneliness, and alleviate depression triggered by stressful events (Denton et al., 2015). High levels of social support are positively associated with increased resilience to frustration, enhanced resilience, and reduced emotional problems. Conversely, lower levels of social support are found to exacerbate negative emotions and are more likely to contribute to the development of depression among adolescents (Fan et al., 2013; Reza et al., 2014). Meanwhile, there is a significant positive correlation between family adaptability and social support (Zhang et al., 2018; Lei and Kantor, 2020). In general, adolescents with high family adaptability are more conducive to enriching emotional communication with others, and in the process of establishing intimate relationships with family members, adolescents can learn how to deal with interpersonal problems properly, which enhances their interpersonal skills and makes them have better interpersonal adaptability in school and social environments (Wang et al., 2020), and the improvement of interpersonal adaptability is conducive to adolescents to gain more social support (Lou et al., 2017). However, a search revealed no literature on social support as a mediator in family adaptability and adolescent depression. Taken together, it is concluded that family adaptability may indirectly influence adolescent depression via social support. Based on these, the following hypothesis is proposed:

*H2*: Social support plays a mediating role between family adaptability and adolescent depression.

### Self-efficacy as a mediator

Self-efficacy is understood as “an individual belief in one’s capabilities to organize and execute the courses of action required in producing given attainments” (Bandura, 1997). In other words, self-efficacy is people’s judgment of their own abilities. According to the ecosystem theory perspective, it is an individual factor that influences adolescent development. Studies have shown that self-efficacy is a potential predictor of adolescent depression (Tore et al., 2018; Li, 2019; Kumar and Rajeev, 2022). Specifically, it is well-known that high self-efficacy can reduce adolescent depression (Moeller and Seehuus, 2019; Tonga et al., 2020). Lau et al. (2022) conducted an experimental study with patients with neurological disorders, they found that self-efficacy was negatively correlated with depression, and that increasing the level of self-efficacy was an effective measure for preventing and intervening in adolescent depression (Lau et al., 2022). In a subsequent study conducted by Volz et al., it was found that self-efficacy negatively predicts the occurrence of depression in adolescents. Moreover, higher levels of self-efficacy were found to effectively decrease the likelihood of behavioral and psychological problems in adolescents, as well as reduce the incidence of depression (Volz et al., 2019). Meanwhile, studies have shown that family adaptability is an important factor influencing self-efficacy, Family adaptability is positively associated with individual self-efficacy (Stubbs, 2015; Yuan et al., 2021). The higher the family adaptability, the higher the adolescents’ pleasure and happiness, and the high positive emotions enable adolescents to enhance their affirmation of their self-efficacy and expect things to go in a good direction (Lee and Shin, 2017), to believe in their ability to get the desired outcome and not to allow themselves to dwell on difficulties or problems (Bandura and Locke, 2003). The findings from the aforementioned studies indicate that there is a clear association between enhanced family resilience and increased self-efficacy. Specifically, the studies suggest that high levels of family adaptability contribute to the establishment of a positive family environment for adolescents. This positive environment not only directly reduces the likelihood of depression in adolescents but also further mitigates depression by fostering an increase in their self-efficacy. However, a search revealed no literature on self-efficacy as a mediator in family adaptability and adolescent depression. Therefore, this study hypothesized that self-efficacy may play a mediating role between family adaptability and adolescent depression. On this basis, the following hypothesis is proposed:

*H3*: Self-efficacy plays a mediating role between family adaptability and adolescent depression.

Orth et al.’s (2016) study provides evidence supporting the notion that social support serves as a robust predictor of positive psychological factors, including self-efficacy, and plays a critical role in safeguarding the mental health of adolescents. Specifically, an increase in social support is associated with higher levels of self-efficacy among individuals (Ostaszewski and Zimmerman, 2006; An et al., 2018; Liu Q. et al., 2020). Social support provides individuals not only with material assistance, but also with psychological support and the promotion of self-efficacy (Adams et al., 2019). That is, individuals with high social support have a higher sense of self-efficacy to combat frustration and reduce the likelihood of depression (Bagci, 2018). Pan (2018) also found that adolescents who have more social support and encouragement were more confident in their ability to get things right when dealing with unexpected situations and new problems and tended to adopt a positive approach to the various problems they faced (Rosa et al., 2022). All of the above studies suggest that social support is an important predictor of self-efficacy. In view of this, the following hypothesis is proposed:

*H4*: Social support and self-efficacy play a chain mediation role between family adaptability and adolescent depression.

**Figure 1:**
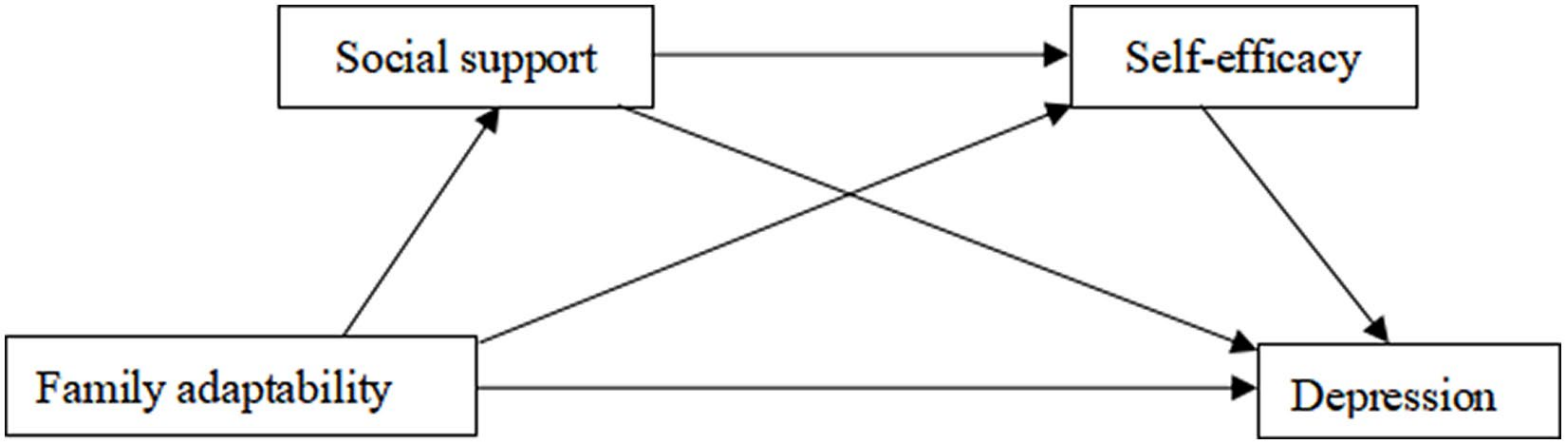
The proposed theoretical model.

In summary, it is proposed that adolescents with elevated levels of family adaptability are more likely to have increased access to essential support and assistance from their family, friends, and society when confronted with challenges. This heightened support provides them with greater confidence to navigate various tasks and challenges, leading to enhanced self-efficacy. Consequently, these factors act as protective mechanisms against the development of depression and other negative emotional states. In light of this, the present study, drawing upon the Ecological Systems Theory, aims to investigate the potential influence of family adaptability on adolescent depression, mediated by the roles of social support and self-efficacy. Moreover, this study explores the potential chain mediating effect of social support and self-efficacy in the relationship between family adaptability and adolescent depression. Based on the aforementioned analysis, the following model is proposed (Figure 1).

## Materials and methods

### Sampling and procedure

A total of 1,143 adolescents were selected as participants for this study through random sampling from seven schools in Shandong Province, China. After excluding the invalid questionnaires with missing answers or consistent responses, 1,086 valid questionnaires were collected, with an effective rate of 95%. Participants were age 12–15 (*M* = 13.72, SD = 0.99). Among them, 465 (42.8%) were boys, and 621 (57.2%) were girls; the valid respondents included 388 (35.7%) in the 6th grade, 182 (16.8%) in the 7th grade, 434 (40.0%) in the 8th grade, and 82 (7.6%) in the 9th grade. The place of residence was urban 628 (57.8%) and rural 458 (42.2%).

### Questionnaire design

#### Family adaptability and cohesion evaluation scale (FACES II-CV)

The measurement of family adaptability was conducted using the Adaptability Subscale of the Family Intimacy and Adaptability Scale, which was revised by Olson (1989). This subscale consists of 14 items, such as “Each member in our family is free to express his or her opinion.” A 5-point Likert scale was used, with scores of 1–5 indicating “hardly ever” to “almost always,” with higher scores indicating higher family adaptability. The scale has good reliability and validity. The Cronbach’s α value of the scale in this study was 0.814. *χ^2^/df* = 4.308, IFI = 0.936, CFI = 0.936, TLI = 0.922, GFI = 0.957, AGFI = 0.940, and RMSEA = 0.055.

#### Perceived social support scale (PSSS)

The Appreciative Social Support Scale (Blumenthal et al., 1987), developed by Zimet et al. (1988), was employed in this study. This scale comprises 12 items. It includes family support (e.g., “I can get emotional help and support from my family when I need it”), friend support (e.g., “My friends share my happiness and sadness”), and other support (e.g., “Some people (leaders, relatives, colleagues) are a real source of comfort for me when I am in trouble”). The 7-point Likert scale was used, with scores ranging from 1–7 indicating “strongly disagree” to “strongly agree,” with higher scores indicating higher perceived social support. The reliability and validity of the scale were good. The Cronbach’s α value of the scale in this study was 0.902, *χ^2^/df* = 7.905, IFI = 0.936, CFI = 0.944, TLI = 0.924, GFI = 0.942, AGFI = 0.908, and RMSEA = 0.080.

#### General self-efficacy scale

The Chinese version of the General Self-Efficacy Scale (GSES) developed by Schwarzer et al. (1997) was used, with 10 questions, such as “I can face difficulties calmly because I can trust my ability to deal with problems.” The scale is scored on a 4-point Likert scale, with scores from 1 to 4 indicating “not at all true” to “completely true,” with higher scores indicating a stronger sense of self-efficacy. The reliability and validity of the scale were good. The Cronbach’s α value of the scale in this study was 0.823, *χ^2^/df* = 8.004, IFI = 0.927, CFI = 0.927, TLI = 0.890, GFI = 0.954, AGFI = 0.915, and RMSEA = 0.080.

#### Center for epidemiological studies depression scale (CES-D)

The Radloff Center for Epidemiological Studies Depression Scale compiled in 1977 was used (Radloff, 1977), with 20 items, such as “I feel that my life is a failure,” of which 4, 8, 12, and 16 are reverse scoring questions, such as “I feel that there is hope for the future.” The scale is used to assess depressed mood or state of mind and focuses more on the individual’s emotional experience and involves fewer somatic symptoms in depression (Hong et al., 2024). The questions were scored on a 4-point scale. 4-point Likert scale was used. The frequency of the corresponding situation or feeling in the past week was evaluated: “not or basically not (less than 1 day)” scored 0, “sometimes or some of the time (1–2 days)” scored 1, “from time to time or half of the time (3–4 days)” was scored as 2, “most or all of the time (5–7 days)” was scored as 3. A total score of ≤15 means no depressive symptoms, 16–19 means possible depressive symptoms, and ≥ 20 means definitely depressive symptoms. The higher the score, the higher the degree of depression. The Cronbach’s α value of the scale in this study was 0.877. *χ^2^/df* = 4.702, IFI = 0.903, CFI = 0.903, TLI = 0.887, GFI = 0.930, AGFI = 0.910, and RMSEA = 0.058.

### Statistical analysis

SPSS 25.0 and AMOS 24.0 were used for statistical analysis. (1) The Harman single-factor test was carried out to test for the common method variance (Tang and Wen, 2020). All items of family adaptability, self-efficacy, social support, and depression were extracted for inclusion in the exploratory factor analysis. The unrotated exploratory factor analysis resulted in a total of 11 factors with a characteristic root greater than 1. The maximum factor variance explained was 20.788%, which is less than the critical value of 40%; therefore, there was no serious common method bias in this study; (2) Descriptive statistics, reliability test and Pearson correlation analysis were carried out on family adaptability, social support, self-efficacy, and depression; (3) The chain mediation test was conducted using AMOS 24.0. Bootstrap in this study was based on 5,000 samples and 95% confidence intervals were generated to test the significance of indirect effects. Bootstrap in this study was based on 5,000 samples and generated the 95% confidence interval to test the significance of indirect effect.

## Results

### Descriptive statistics and correlation analyses

The mean and standard deviation of each variable and the results of the correlation matrix between the variables are detailed in Table 1.

**Table 1:** Descriptive statistics and correlation analysis (*N* = 1,086).

	*M*	SD	1	2	3	4
1 Family adaptability	46.866	9.744	1			
2 Social support	62.579	12.933	0.507^**^	1		
3 self-efficacy	25.971	5.417	0.376^**^	0.348^**^	1	
4 Depression	14.923	9.224	−0.407^**^	−0.360^**^	−0.298^**^	1

^*^p < 0. 05, ^**^p < 0.01, ^***^p < 0. 001, same below.

As shown in Table 1, their pathways were supported by the data. Specifically, family adaptability and adolescent depression established a significant negative relationship (*r* = −0.407, *p* < 0.01); family adaptability and social support established a significant positive relationship (*r* = 0.507, *p* < 0.01); social support was significantly negatively related to adolescent depression (*r* = −0.360, *p* < 0.01); family adaptability was significantly and positively related to self-efficacy (*r* = 0.376, *p* < 0.01); self-efficacy was significantly negatively related to adolescent depression (*r* = −0.298, *p* < 0.01); and social support significantly and positively related to self-efficacy (*r* = 0.348, *p* < 0.01). The relationship between the variables supports the subsequent hypothesis testing.

### Testing for mediation effect

To analyze the mediating effect, AMOS is used to test the mediating effect. The bootstrap proposed by MacKinnon (2012) was used for significance testing, with a sample size of 5,000 and a confidence level of 95%. The model was fitted by AMOS, the fitting index of the model was *χ^2^*/df = 5.411, IFI = 0.990, CFI = 0.990, TLI = 0.970, GFI = 0.992, AGFI = 0.966, and RMSEA = 0.064. Each index is in an acceptable range, and the model is ideal.

The significance of the mediating effect was tested using the nonparametric percentage Bootstrap procedure with bias correction, with 5,000 repetitive samples and 95% confidence intervals calculated. Accordingly, the results of the mediating effect of social support and self-efficacy between family adaptability and adolescent depression were analyzed (see Table 2 for details). The total effect value was −0.385. The direct effect value of family adaptability on adolescent depression was −0.251. Hypothesis 1 was established. The total indirect mediating effect size for social support and self-efficacy was −0.134 with a 95% confidence interval of [−0.179, −0,095], which does not contain 0, indicating that there is a significant mediating effect of social support and self-efficacy between family adaptability and depression. This total indirect mediating effect arose through three mediating paths: the first indirect effect Ind1: family adaptability → social support → depression had an indirect effect of −0.085 with a 95% confidence interval of [−0.122, −0.051], which did not contain 0, indicating that social support mediated a significant effect between family adaptability and depression. Hypothesis 2 was established. The second indirect effect Ind2 had a path of family adaptability → self-efficacy → depression, with an effect size of −0.035 and a 95% confidence interval of [−0.057,−0.018], which does not contain 0, indicating that self-efficacy mediates a significant effect between family adaptability and depression. Hypothesis 3 was established. The third mediated path Ind3 is: family adaptability → social support → self-efficacy → depression, with a mediated effect size of −0.014 and a 95% The confidence interval was [−0.024,−0.007] not containing 0, indicating that the chain mediating effect of social support and self-efficacy between family adaptability and depression was significant. Hypothesis 4 was established.

**Table 2:** Mediating effects between family adaptability, self-efficacy, social support and depression variables (*N* = 1,086).

	Effect	Boot SE	Boot LLCI	Boot ULCI	The proportion of total indirect effect
Total effect	−0.385	0.018	−0.442	−0.331	
Direct effect	−0.251	0.021	−0.317	−0.182	
Total indirect effect	−0.134	0.010	−0.179	−0.095	
Ind1	−0.085	0.004	−0.122	−0.051	63.7%
Ind2	−0.035	0.034	−0.057	−0.018	25.9%
Ind3	−0.014	0.028	−0.024	−0.007	10.4%
Ind1 − Ind2	−0.051	0.022	−0.094	−0.007	
Ind1 − Ind3	−0.072	0.019	−0.109	−0.035	
Ind2 − Ind3	−0.021	0.009	−0.043	−0.007	

Boot SE, Boot LLCI and Boot ULCL refer to the standard error, lower and upper limits of the 95% confidence interval of the indirect effects estimated by the bias-corrected percentile Bootstrap method, respectively.

**Figure 2:**
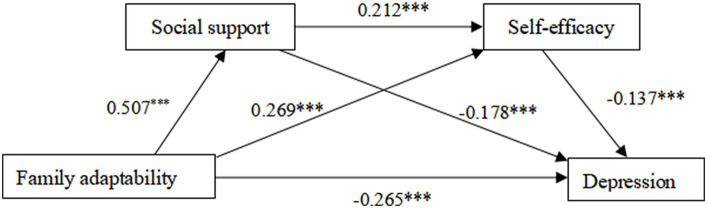
The chain mediation model.

The specific pathways of family adaptation acting on depression through social support and self-efficacy are detailed in Figure 2.

Differences between the three chained mediated pathways were compared, and the data results are detailed in Table 2. The difference between indirect effect 1 and indirect effect 2 was significant, Bootstrap did not contain 0 at the 95% confidence interval [−0.094, −0.007], and the mediated effect from social support alone (63.7%) was higher than the effect from self-efficacy (25.9%). The difference between indirect effect 1 and the difference between indirect effect 3 was significant, Bootstrap did not contain 0 at the 95% confidence interval [−0.109, −0.035], and the mediating effect from social support alone (63.7%) was higher than the effect from social support and self-efficacy together (10.4%). The difference between indirect effect 2 and indirect effect 3 was significant, Bootstrap at the 95% confidence interval [−0.043, −0.007] did not contain 0, and the mediating effect of self-efficacy alone (25.9%) was higher than the effect of social support and self-efficacy together (10.4%). In conclusion, social support mediated the relationship between family adjustment and depression (63.7%), with a greater effect than the mediating effect of self-efficacy (25.9%) and the chain mediating effect of social support and self-efficacy (10.4%).

## Discussion

Depression, as an important component of adolescent mental health, has become increasingly important to the healthy growth of adolescents, and its research has received more and more attention. Depression not only has a negative impact on daily life, learning and social adjustment, but also aggravates the psychological burden of adolescents (Tang et al., 2019). However, existing studies have mainly explored the influencing factors of adolescent depression, and have paid less attention to the formation mechanisms of adolescent depression. Therefore, based on the Ecological Systems Theory, this study constructs a model of family adaptability, social support, self-efficacy and adolescent depression, and explores the effects and pathways of family adaptability, social support and self-efficacy on adolescent depression. The model helps us understand how and under what conditions family adaptability affects adolescent depression, providing potential guidance for improving levels of adolescent depression and enhancing adolescent mental health.

### The relationship between family adaptation and adolescent depression

As shown in Table 1, the correlation between family adaptability and depression is 0.407, which is a moderate correlation and significant at the 0.01 level. The findings suggest that family adaptability predicts adolescent depression with an effect size of −0.251 (Table 2). H1 was valid, which is consistent with the results of previous studies (Wang et al., 2021). The predictive effect remained significant after the inclusion of mediating variables. That is, the less well-adjusted the adolescent is in the family, the more likely he or she is to show emotional problems such as anxiety and depression (Li et al., 2021; Ying et al., 2022). This may be due to the fact that adolescents with low family adaptability lack the necessary emotional communication and support in the family, which not only makes the adolescents less adaptable in facing problems and challenges, but also predisposes the adolescents to feel more stressed and thus depressed (Berryhill and Smith, 2021). In summary, the results of this study further underscore that family adaptability can significantly and negatively predict depression in adolescents. The findings also corroborate the applicability of ecosystem theory and its theoretical guidance in the Chinese family education context.

### The mediating role of social support

The findings suggest that social support plays a mediating role between family adaptability and adolescent depression with an effect size of −0.085 (Table 2). H2 was valid, which is consistent with He et al.’s (2021) study that family adaptability is significantly and positively associated with social support. Individuals with high family adaptability have clear roles for their family members. Everyone can perform their own duties, cooperate with each other, and listen to the opinions of each member when they encounter problems. And they can adjust the family environment and change certain family roles at any time to cope with family changes. This allows individuals to feel loved and wanted in their families. At the same time, they have family members to help them when they encounter difficulties, thus enhancing the level of family support for individuals. (Jewell et al., 2015). The findings of this study shows that social support is significantly and negatively associated with adolescent depression, which is consistent with previous studies (Davidson and Adams, 2013; Wang et al., 2018). Social support does have an important role in relieving adolescents’ stress, contributing to stabilizing their emotional state and having a positive impact in promoting adolescents’ mental health (Qi et al., 2020; Ringdal et al., 2020). For adolescents, social support has a transitional role when they face crises, making them feel more trusted, respected, tolerant, understanding, and concerned, and less anxious and depressed (Xing et al., 2016; Kang et al., 2022). The present study enriches the explanatory mechanism of family adaptability affecting adolescent depression from the perspective of ecological systems theory. Since the family is the most direct environmental system of an individual and society is the largest environmental system affecting an individual, increased family adaptability can increase social support, and increased social support can effectively reduce the occurrence of depression. In summary, the results of this study further underscore the significance of family adaptability as a negative and significant predictor of depression in adolescents.

### The mediating role of self-efficacy

The findings suggest that self-efficacy serves as a significant partial mediating variable in the relationship between family adaptability and adolescent depression with an effect size of −0.035 (Table 2). H3 was valid. In the present study, family adaptability was found to be positively associated with self-efficacy, a finding that is consistent with previous studies (Yuan et al., 2021; Jackson et al., 2022). Adolescents who experience high levels of family resilience tend to possess enhanced problem-solving skills and confidence in their decision-making abilities when faced with challenges, thus bolstering their self-efficacy (Ali et al., 2020). Additionally, Li, 2021 also found that high family resilience ensures that adolescents receive more life care and emotional support during their development, which helps to regulate their psychology. It helps adolescents to establish good psychological adaptability strategies and role adaptation, thus enhancing their ability to cope with difficulties and directly improving their self-efficacy (Li, 2021). Moreover, self-efficacy has been found to influence individuals’ attitudes toward learning, as well as their cognition and emotions (Xu and Li, 2021; Liu et al., 2023). Further, self-efficacy is significantly and negatively associated with adolescent depression (De et al., 2018; Maurya et al., 2023), and the same results are found in this paper. Self-efficacy plays a key role in an individuals’ emotion regulation process, which helps them to maintain a high level of self-confidence when facing problematic situations, and this enables them to cope with stress effectively, manage their negative emotions better, and reduce the frequency and duration of depression (Tang et al., 2010; Bandura, 2012; Liu D. et al., 2020; Wen et al., 2021). Individuals with high self-efficacy have positive attitudes toward themselves and are more inclined to view external setbacks and difficulties as temporary, which helps to reduce depression caused by psychological stress (Pu et al., 2017; Li, 2019). In summary, self-efficacy serves as another crucial pathway through which family adaptability affects adolescent depression.

### The chain mediating role of social support and self-efficacy

Another important finding of this study is that family adaptability can influence the onset of depression in adolescents through a chain mediating effect of social support and self-efficacy. The effect value of the chain mediated path is −0.014 (Table 2). H4 was valid. Specifically, social support was found to positively predict self-efficacy, which aligns with previous research (Adams et al., 2019; Liu Q. et al., 2020). Social psychology suggests that the social environment provides individuals with emotional support, instrumental support, and evaluative support, which facilitates the continuous reconstruction and refinement of personal beliefs, ultimately enhancing individual self-efficacy (Zhou et al., 2014). Moreover, a higher sense of self-efficacy has been consistently linked to a reduction in depression among individuals, as supported by previous studies (Bagci, 2018; Lee et al., 2020). In summary, family adaptability can enhance self-efficacy via social support, thereby reducing the likelihood of adolescent depression. Overall, this study analyzes the complex relationship among family adaptability, social support, self-efficacy and adolescent depression based on Ecological Systems Theory, which enriches the research related to adolescent depression to some extent.

### The theoretical and practical implications

This study holds several theoretical and practical implications. From a theoretical standpoint, it contributes to the existing literature on factors influencing adolescent depression. Specifically, it establishes a negative association between family adaptability and adolescent depression, highlighting that higher levels of family adaptability are associated with better mental health outcomes and reduced likelihood of depression among adolescents (Jia and Yuan, 2018). This enriches the literature on factors influencing adolescent depression. Furthermore, this study shows that social support and self-efficacy influence the association between family adaptability and adolescent depression through the role of chain mediators, contributing to the understanding of the mechanisms of family adaptability on adolescent depression. Adolescents with high family adaptability can improve social support and develop their self-efficacy, thus reducing adolescents’ depression. From a practical perspective, this study provides directions for preventing and reducing adolescent depression in terms of environmental factors (family adaptability, social support) as well as individual factors (self-efficacy). In terms of family adaptability, family members should help each other, communicate and understand each other when they encounter problems, and establish a democratic family atmosphere; at the same time, family members should negotiate together to establish relatively clear internal family norms, each doing his or her own job, and establishing a strong internal organization. In terms of social support, parents and teachers should not only focus on students’ grades but also on other needs of adolescents and provide corresponding encouragement and support; not only material but emotional support and assistance should be given to meet various psychological needs of adolescents and help them to build a well-established social support system; at the same time, schools can adopt scientific means to conduct regular surveys on students’ mental health, and adopt scientific methods to regularly screen students’ mental health and intervene early for students who are screened for possible anxiety or depression to reduce the likelihood of depression. On the one hand, schools should design activities according to the characteristics of adolescents, set up more incentives to encourage students to participate in them, provide them with opportunities and platforms to show themselves, and increase their chances of success, so as to enhance their sense of self-efficacy. On the other hand, parents should be good at recognizing their children’s sparkling points and give them correct guidance, positive encouragement and timely feedback to continuously strengthen their sense of self-efficacy. At the same time, adolescents should also make full use of the “role model effect” and set reasonable goals for themselves to maintain their self-belief and improve their self-efficacy.

### Limitations and future research directions

While this study has provided insights into the internal mechanisms of family adaptability and adolescent depression, there are several limitations that can be addressed in future research to further enhance the understanding in this area. Due to the self-reporting approach used in this study, the following problems existed: (1) This study only used cross-sectional research, which is a correlational study and cannot explore the causal relationship between variables and reflect the continuous and stable relationship between variables, and future follow-up surveys can be conducted on some families to longitudinally to explore the influence of family adaptability on adolescent depression, in order to improve the external validity of the study. (2) The sample of this study was taken from only seven middle schools in a province in northern China, and the ecological validity of the results may be low. Therefore, the results of this study cannot be easily generalized to southern China and other cultural contexts. In the future, the sample can be enlarged for re-administration or cross-cultural studies can be attempted. (3) There are many other factors that can influence adolescent depression, such as family caregiving, self-esteem, and so on, and more variables can be used to produce more comprehensive and practically meaningful results in future studies. By exploring the interactions and combined effects of various factors, a more holistic understanding of the complex nature of adolescent depression can be achieved.

## Data availability statement

The raw data supporting the conclusions of this article will be made available by the authors, without undue reservation.

## Ethics statement

The studies involving humans were approved by Biomedical Ethics Committee of Jining University. The studies were conducted in accordance with the local legislation and institutional requirements. Written informed consent for participation in this study was provided by the participants’ legal guardians/next of kin.

## Author contributions

YL: Writing – original draft, Writing – review & editing. GJ: Writing – review & editing, Writing – original draft. ZZ: Writing – review & editing, Writing – original draft. ML: Writing – review & editing. GC: Writing – review & editing, Writing – original draft.
